# SARS-CoV-2 Seroprevalence and Neutralizing Antibody Response after the First and Second COVID-19 Pandemic Wave in Croatia

**DOI:** 10.3390/pathogens10060774

**Published:** 2021-06-20

**Authors:** Tatjana Vilibic-Cavlek, Vladimir Stevanovic, Maja Ilic, Ljubo Barbic, Krunoslav Capak, Irena Tabain, Jasna Lenicek Krleza, Thomas Ferenc, Zeljka Hruskar, Renata Zrinski Topic, Vanja Kaliterna, Arlen Antolovic-Pozgain, Jasmina Kucinar, Iva Koscak, Dijana Mayer, Mario Sviben, Ljiljana Antolasic, Ljiljana Milasincic, Lovro Bucic, Ivana Ferencak, Bernard Kaic

**Affiliations:** 1 Department of Virology, Croatian Institute of Public Health, 10000 Zagreb, Croatia; irena.tabain@hzjz.hr (I.T.); zeljka.hruskar@hzjz.hr (Z.H.); ljiljana.antolasic@hzjz.hr (L.A.); ljiljana.milasincic@hzjz.hr (L.M.); ivana.ferencak@hzjz.hr (I.F.); 2 Department of Microbiology, School of Medicine, University of Zagreb, 10000 Zagreb, Croatia; mario.sviben@hzjz.hr; 3 Department of Microbiology and Infectious Diseases with Clinic, Faculty of Veterinary Medicine, University of Zagreb, 10000 Zagreb, Croatia; vladostevanovic@gmail.com (V.S.); ljubo.barbic@vef.hr (L.B.); 4 Department of Epidemiology, Croatian Institute of Public Health, 10000 Zagreb, Croatia; maja.ilic@hzjz.hr (M.I.); lovro.bucic@hzjz.hr (L.B.); bernard.kaic@hzjz.hr (B.K.); 5 Environmental Health Department, Croatian Institute of Public Health, 10000 Zagreb, Croatia; kcapak@hzjz.hr; 6 Department of Laboratory Diagnostics, Children’s Hospital Zagreb, 10000 Zagreb, Croatia; jlenicek@gmail.com (J.L.K.); renata.zrinski-topic@zg.t-com.hr (R.Z.T.); 7 Clinical Department of Diagnostic and Interventional Radiology, Merkur University Hospital, 10000 Zagreb, Croatia; thomas.ferenc95@gmail.com; 8 Department of Clinical Microbiology, Teaching Institute of Public Health of Split-Dalmatia County, 21000 Split, Croatia; vanja.kaliterna@gmail.com; 9 Department of Microbiology, Osijek-Baranja County Institute of Public Health, 31000 Osijek, Croatia; arlenpozgain@gmail.com; 10 Department of Serology, Istria County Institute of Public Health, 52100 Pula, Croatia; jasmina.kuci-nar@zzjziz.hr; 11 Department of Microbiology, Varazdin County Institute of Public Health, 42000 Varazdin, Croatia; iva.koscak@vz.t-com.hr; 12 Department for Monitoring and Improving of School and Youth Health, Croatian Institute of Public Health, 10000 Zagreb, Croatia; dijana.mayer@hzjz.hr; 13 Department of Parasitology, Croatian Institute of Public Health, 10000 Zagreb, Croatia

**Keywords:** COVID-19, SARS-CoV-2, seroprevalence, ELISA, VNT, Croatia

## Abstract

Severe acute respiratory syndrome coronavirus 2 (SARS-CoV-2) is a novel coronavirus with a pandemic spread. So far, a total of 349,910 SARS-CoV-2 cases and 7687 deaths were reported in Croatia. We analyzed the seroprevalence and neutralizing (NT) antibody response in the Croatian general population after the first (May–July 2020) and second (December 2020–February 2021) pandemic wave. Initial serological testing was performed using a commercial ELISA, with confirmation of reactive samples by a virus neutralization test (VNT). A significant difference in the overall seroprevalence rate was found after the first (ELISA 2.2%, VNT 0.2%) and second waves (ELISA 25.1%, VNT 18.7%). Seropositive individuals were detected in all age groups, with significant differences according to age. The lowest prevalence of NT antibodies was documented in the youngest (<10 years; 16.1%) and the oldest (60–69/70+ years; 16.0% and 12.8%, respectively) age groups. However, these age groups showed the highest median NT titers (32–64). In other groups, seropositivity varied from 19.3% to 21.5%. A significant weak positive correlation between binding antibody level as detected by ELISA and VNT titer (rho = 0.439, *p* < 0.001) was observed. SARS-CoV-2 NT antibody titers seem to be age-related, with the highest NT activity in children under 10 years and individuals above 50 years.

## 1. Introduction

Coronavirus disease (COVID-19) is caused by a novel severe respiratory syndrome coronavirus 2 (SARS-CoV-2) that emerged in late 2019 in Wuhan, China. SARS-CoV-2 is a highly transmissible coronavirus. The international spread accelerated from late February 2020, with large clusters of cases reported from an increasing number of countries [1]. On 30 January 2020, the World Health Organization declared the COVID-19 outbreak a public health emergency of international concern [2], and on 11 March 2020, a pandemic was declared [3]. 

The high transmissibility of SARS-CoV-2 may be attributed to the patterns of virus shedding. The SARS-CoV-2 viral load in upper respiratory tract samples is highest during the first week of symptoms, and thus the risk of pharyngeal virus shedding is very high at the beginning of infection [1]. Some viral variants, such as the variant B.1.1.7, first identified in the United Kingdom, as well as variant B.1.351 and variant P.1, first identified in South African and in Brazilian travelers, respectively, seem to spread more efficiently and rapidly than other variants. Although there is no clear evidence for any impact on disease severity, enhanced transmission will lead to higher incidence, more hospital admissions and potentially more deaths [4,5,6]. Persons with asymptomatic infection (20–75% of COVID-19 cases, according to different studies) can also transmit the virus [7]. 

The clinical spectrum of SARS-CoV-2 infection in humans varies from asymptomatic or mild symptoms (81%) to severe respiratory failure (14%) and critical disease (5%). Older patients (>60 years) and those with pre-existing diseases have a greater risk of developing severe disease. The rapid virus replication in the lungs may trigger a strong immune response. Cytokine storm syndrome causes acute respiratory distress syndrome and respiratory failure, which is considered the main cause of death in patients with COVID-19, according to some authors [1]. With the exception of multisystem inflammatory syndrome in children (MIS-C), pediatric and adolescent populations usually have a milder form of the disease [8]. 

In Croatia, the first case of COVID-19 was reported on 25 February 2020. In March 2020, restrictive epidemiological measures were introduced (initial lockdown) to inhibit the disease’s spread. With the gradual relaxing of restrictive measures in May and during the summer, the number of COVID-19 cases gradually increased, while in September and October, this number increased sharply [9]. In February 2021, the first cases of COVID-19 caused by the B.1.1.7 variant were detected. Sequencing results showed a marked continuous increase in the B.1.1.7 presence, from 21% in the first week of February to 96% in the last week of April. This increase in the ratio of B.1.1.7 in all sequenced samples follows the rise of daily positive COVID-19 cases and the ascending curve of the third pandemic wave in Croatia. COVID-19 patients were recorded in all Croatian counties, with a total of 358,581 cases and 8152 deaths reported as of 14 June 2021 [10].

Several seroprevalence studies were conducted after the first pandemic wave, in specific population groups showing low seropositivity (1.27–5.19%) [11,12,13]. The prevalence of neutralizing (NT) antibodies was even lower (<1%) [12]. A more recent study conducted among the pediatric population from the Children’s Hospital Zagreb has shown an increase in the prevalence of NT antibodies, from 2.9% after the first wave (May 2020) to 8.4% at the peak of the second wave (October–November 2020). This difference was expected due to increased COVID-19 incidence in the country [9]. However, there are no data on the seroprevalence in the general population as well as the NT antibody response profiles to SARS-CoV-2 in neither the pediatric nor adult Croatian population.

The aim of this study was to analyze the seroprevalence and NT antibody titers in a large sample of the Croatian general population after the first and second COVID-19 pandemic wave.

## 2. Results

**Figure 1:**
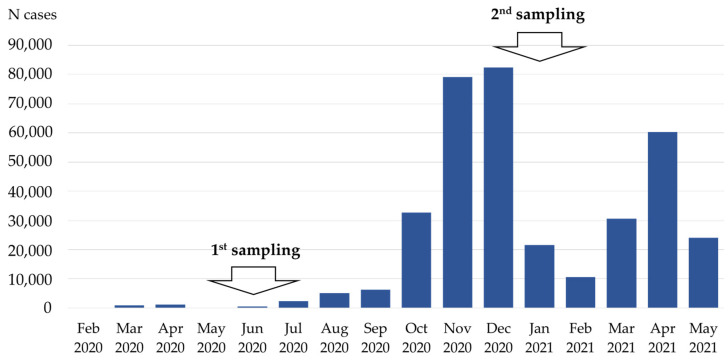
COVID-19 epidemic curve in Croatia. During the first (February–July 2020) and second (August 2020–February 2021) pandemic wave, 5101 and 237,835 COVID-19 cases, respectively, were reported in Croatia. The introduction of the B.1.1.7 variant in February 2021 that spread rapidly resulted in a third pandemic wave, with 113,168 COVID-19 cases.

Three COVID-19 epidemic waves were reported in Croatia (Figure 1).

SARS-CoV-2 seroprevalence results are presented in Table 1. After the first wave, 24/1088 (2.2%) participants showed IgG antibodies using enzyme-linked immunosorbent assay (ELISA), while only two participants (0.2%) had NT titers ≥ 8. A significantly higher overall seroprevalence rate was detected after the second wave. SARS-CoV-2 IgG antibodies were detected in 360/1436 (25.1%) participants, and NT antibodies (titers ≥ 8) were confirmed in 268 (18.7%) participants (*p* < 0.001). In addition, a significantly higher prevalence rate of NT antibodies was detected in ELISA-positive participants detected during the second wave (268/360; 74.4%) compared to the first wave (2/24; 8.3%) (*p* < 0.001).

Due to the small number of seropositive participants detected at the first time-point sampling, the association of SARS-CoV-2 seroprevalence rates and antibody response with patients’ demographic characteristics was analyzed only for participants tested at the second time-point. NT seroprevalence rates did not differ between males and females (19.1% vs. 17.7%; χ^2^ = 2.803, *p* = 0.094). However, there was a significant difference in seropositivity between age groups (χ^2^ = 14.891, *p* = 0.037). The lowest NT seroprevalence rates were recorded in the oldest age groups (12.8% and 16.0% in patients 70+ years and 60–69 years, respectively) followed by the youngest (<10 years) age group (16.1%). In other groups, seropositivity varied from 19.3% to 21.5%.

**Figure 2:**
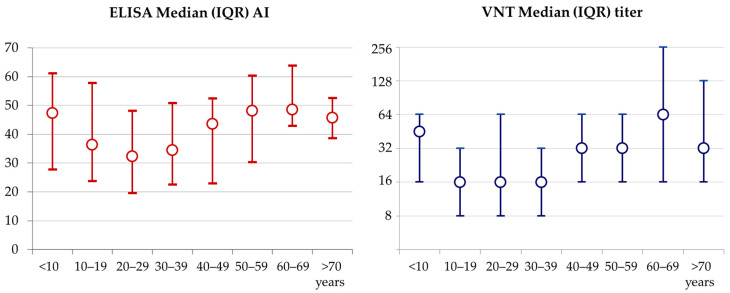
AI (ELISA) and NT antibody titer in SARS-CoV-2 seropositive participants by age group. Children < 10 years and patients > 60 years showed both higher median AI and VNT titer compared to other age groups.

The SARS-CoV-2 serological response in seropositive participants, according to patients’ age, is presented in Figure 2. The highest median of binding AI and NT antibody titers were detected in the participants >50 years, as well as in participants <10 years. In the age groups 50–59, 60–69 and 70+ years, the median AI was 48.1 (IQR = 30.3–60.3), 48.6 (IQR = 42.9–63.8), and 45.7 (IQR = 38.6–52.6), respectively. The median NT titer was 32 (IQR = 16–64), 64 (IQR = 16–256) and 32 (IQR = 16–128), in the respective age groups. In children under 10 years, the median AI was 47.3 (IQR = 27.8–61.1), while the median NT titer was 48 (IQR = 16–64). Patients aged 10–39 years exhibited both the lowest median IgG AI (32.3–43.6) and NT titer (16–32). These differences were statistically significant (Kruskal–Wallis test AI *p* = 0.025; NT *p* = 0.001).

**Figure 3:**
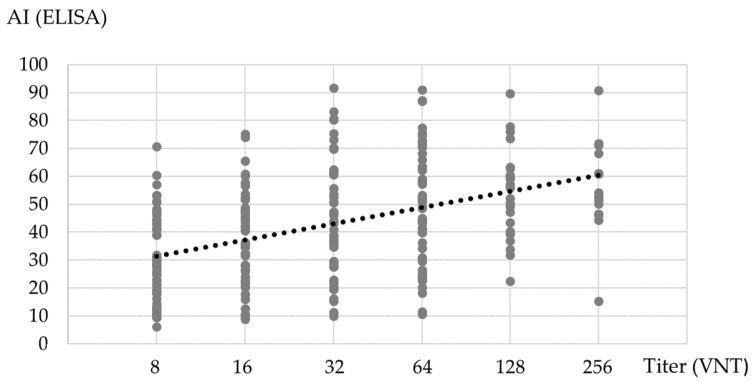
Correlation of binding AI and VNT titer. The levels of NT antibodies showed a weak positive correlation with the levels of binding SARS-CoV-2 IgG antibodies.

A significant weak positive correlation between binding AI (ELISA) and VNT titers was observed (Spearman’s rho = 0.439, *p* < 0.001) (Figure 3).

**Figure 4:**
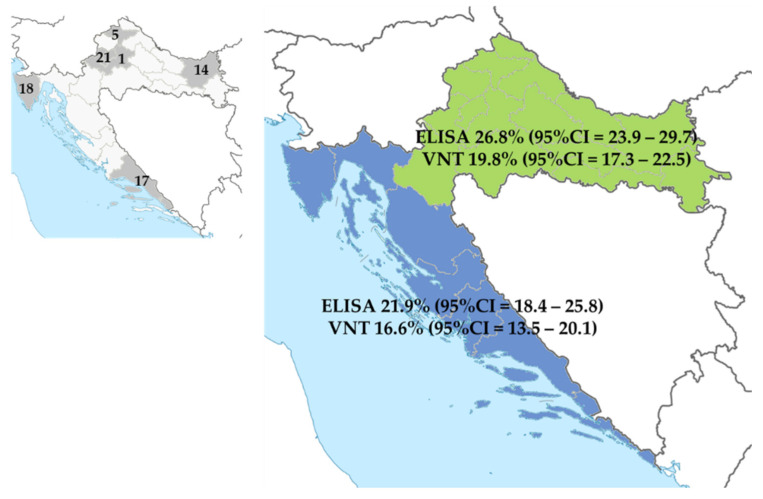
SARS-CoV-2 seroprevalence by geographic region. The prevalence of SARS-CoV-2 binding antibodies differed significantly between regions and was higher in continental counties. The prevalence of NT antibodies was higher in the inhabitants of continental counties; however, this difference was not significant.

To determine the seroprevalence according to geographic region, seropositivity was analyzed in six selected distant counties (four continental and two coastal). The overall SARS-CoV-2 seroprevalence rates differed significantly between the continental and coastal regions in the ELISA test (249/930; 26.8% vs. 111/506; 21.9%, Pearson χ^2^ = 4.082, *p* = 0.043). However, there was no significant difference in the prevalence of NT antibodies (184/930; 19.8% vs. 84/506; 16.6%, Pearson χ^2^ = 2.188, *p* = 0.139) (Figure 4).

Seroprevalence analysis by counties showed significant differences in the seropositivity in both ELISA (Pearson χ^2^ = 26.472, *p* < 0.001) and VNT (Pearson χ^2^ = 23.499, *p* < 0.001) (Table 2).

## 3. Discussion

The results of this first large seroprevalence study in the Croatian general population (N = 2524) showed a significant difference in the SARS-CoV-2 seropositivity rates after the first (May-July 2020) and second pandemic wave (December 2020–February 2021). Using ELISA, SARS-CoV-2 IgG antibodies were detected in 2.2% and 25.1% of participants, respectively. NT antibodies were found in only 0.2% of participants tested after the first wave compared to 18.7% of participants after the second wave. It is important to note that the prevalence of NT antibodies in ELISA-positive individuals was significantly higher after the second wave (74.4%) compared to only 8.3% after the first wave. 

Several published studies have reported the SARS-CoV-2 seroprevalence in Croatia after the first wave in specific population groups. In April 2020, 1494 factory employees living in two counties at the Croatian littoral (Split-Dalmatia and Sibenik-Knin) were tested for the presence of SARS-CoV-2 antibodies. Using a rapid immunochromatographic test, IgG antibodies were detected in 1.27% of participants [11]. In addition, during April and May 2020, a seroprevalence study on the SARS-CoV-2 among personnel in the healthcare facilities of Croatia was performed. A total of 592 serum samples from healthcare workers (HCW) and allied/auxiliary HCW were tested for the presence of SARS-CoV-2 antibodies. Convenient samples were collected from six counties with a high incidence of COVID-19. Using ELISA, IgG antibodies were detected in 16 (2.7%) participants, while NT antibodies (titers 32 to 256) were confirmed in 9 (1.5%) participants [12]. Moreover, in May 2020, 122 serum samples from employees of the Faculty of Veterinary Medicine University of Zagreb were tested. A total of 5.19% of the administrative, basic and pre-clinical sciences department personnel and 5.13% of the animal health service providers and laboratory personnel tested ELISA-positive; however, NT antibodies were not detected in any sample [13]. One more recent study analyzed the prevalence of antibodies among the pediatric population in the Children’s Hospital Zagreb at two time points. The overall SARS-CoV-2 seropositivity was 6.0% (33/548), with significantly lower seroprevalence (2.9%) after the first wave (May 2020) compared to the seroprevalence at the peak of the second wave (8.4%) (October–November 2020) [9]. In addition, from September to November 2020 (the beginning of the second wave), a cross-sectional screening for COVID-19 in the adult outpatient liver (N = 280) and kidney transplant recipients (N = 232) was performed. The transplanted cohort’s seroprevalence was 20.1%, and 15.6% of anti-SARS-CoV-2 ELISA IgG-positive patients developed NT antibodies [14].

As in Croatia, seroprevalence rates in the general population across Europe after the first pandemic peak were low: 0.36% in Greece (March–April 2020) [15], 1.9–2.9% in the Czech Republic (May 2020) [16], 4.3% in Scotland (June 2020) [17], 4.5% in the Netherlands (June–August 2020) [18] and 4.6% in Spain (April–May 2020) [19]. In Germany (April–June 2020), seropositivity was 0.97% using ELISA, and 0.36% using VNT [20]. Significantly higher seropositivity was observed after the second pandemic peak, varying from 11.4% in Poland (October–November 2020) [21], and 19.3% in Spain (Madrid, at the end of 2020) [22], to 21.1% in Switzerland (Geneva, November–December 2020) [23]. In Slovenia, the seroprevalence after the first (April 2020) and second (October–November 2020) waves was found to be 2.78% and 4.06%, respectively [24].

The results of this study showed that COVID-19 occurred in all age groups. However, there were significant differences in the NT-antibody positivity rates, from 12.8% to 21.5%. The lowest seropositivity was documented in the oldest (12.8–16.0%) and youngest (16.1%) age groups, compared to 19.3%-21.5% in other groups. A similar age distribution of SARS-CoV-2 infections was detected in a large US study, conducted during the convalescent stage of previously asymptomatic or mildly ill non-hospitalized pediatric and adult patients. Although the overall positivity rates for pediatric and adult individuals were not significantly different (16.5% vs. 18.6%), positivity rates differed among different age groups. Children aged 1–10 years and patients aged 60+ years showed the lowest seroprevalence rates (15.2% and 12.9–15.9%, respectively), while young adults (19–30 years) had the highest seropositivity (23.5%–24.4%) [25]. Similarly, large differences in seropositivity across age groups were detected in Switzerland. Compared with adults aged 25–34 years, children older than 6 years and adolescents had similar seroprevalence, whereas children aged 0–5 years were 43% less likely to be seropositive. Additionally, adults aged 65–74 years, and those older than 75 years, were 42% and 64% less likely to be SARS-CoV-2 seropositive [23]. Children and the elderly were probably less exposed to SARS-CoV-2 compared to young adults and the working-age population. Furthermore, immunosenescence in both innate and adaptive immune systems could contribute to a lower seroprevalence in the elderly [26]. Children appear to be less susceptible to SARS-CoV-2 infection. There have been far fewer confirmed cases of COVID-19 disease in children (children consistently make up 1–5% of total case numbers in reports) [27,28,29]. In South Korea, children under nine years accounted for only 1% of laboratory-confirmed cases of COVID-19, whereas children aged 10–19 years accounted for 5.2% of cases [30]. Similarly, in Iceland, young children were less likely to test positive for SARS-CoV-2 than were adolescents or adults [31]. Since children often experience milder symptoms than adults, they are less likely to be tested, which could be one possible explanation for these low numbers. Additionally, a large proportion of children with SARS-CoV-2 infection are asymptomatic [32]. 

Some evidence suggests that SARS-CoV-2 antibody responses may differ in children and adults; however, the physiological mechanisms for these differences are unclear [25,33]. Very few studies analyzed the antibody response to SARS-CoV-2 in the pediatric and adult populations. The results of this study showed that children under 10 years of age showed significantly higher median SARS-CoV-2 IgG AI, as well as SARS-CoV-2 NT antibody titer, compared to adolescents and young adults. Additionally, median binding AI and NT titer were found to be higher in participants older than 50 years. Several studies showed that SARS-CoV-2 higher NT antibody titers were induced in the severely ill group as compared with mildly ill patients. Since aging itself is a prominent risk factor for severe disease and death from COVID-19, higher NT antibody titers in the elderly could be explained by more severe symptoms in this population group [34,35]. An age-associated seroprevalence similar to our results was observed in the US study. The IgG level in the pediatric population showed a moderate, but significant, negative correlation with age, while the adult population showed a weakly positive correlation with age. Children aged 1 to 10 years had significantly higher median SARS-CoV-2 IgG levels than adolescents aged 11 to 18 years. Similarly, an inverse correlation of surrogate NT antibody activity with age was found [25]. A possible explanation for higher antibody levels in children is cross-reactivity with seasonal human coronaviruses, since epitopes for T and B cells were found to be conserved among SARS-CoV-2, HCoV-OC43 and HKU1 [36]. Higher NT antibody titers in children could be responsible for their milder COVID-19 symptoms [25]. Like in the US study, our results showed that adolescents and young adults exhibited lower median NT antibody titers (16) compared to other age groups (32–64); however, this difference remains unclear. Young adults have high rates of asymptomatic and mild infections, which could be associated with lower levels of antibodies.

In contrast, the other US study showed a positive correlation of NT antibody titer with age in hospitalized children and adults with COVID-19. Significantly lower NT antibody activity was detected in two subgroups of pediatric/young adult patients (<24 years, without mechanical ventilation and MIS-C) [37]. No differences in the in vitro virus neutralization were observed between the different age groups of Australian COVID-19 patients. However, higher cross-reactive SARS-CoV-2 IgA and IgG antibodies were observed in healthy elderly people, while healthy children display elevated SARS-CoV-2 IgM, suggesting that children have had fewer human coronavirus exposures, resulting in less experienced but broadly reactive humoral immunity [38].

Comparing the antibody levels in ELISA and VNT, our results showed a weak positive correlation of SARS-CoV-2 binding IgG antibodies (ELISA) and NT antibody titers. A Chinese study found similar results, i.e., moderate correlation of anti–SARS-CoV-2 S and N IgG with NT titers, suggesting that monitoring S and N antibody levels could be useful as an indirect indicator of neutralizing activity [34].

The results of this study showed a significant difference in seropositivity among regions, with a higher overall seroprevalence rate in continental Croatian counties compared to coastal counties. This geographic difference is expected, due to the great differences in reported incidences of COVID-19 by region.

This study indicates that a large proportion of the population is still susceptible to COVID-19, and that the end of the epidemic cannot be expected in the near future due to population immunity conferred by natural infection. In order to prevent thousands of deaths in future epidemic waves, population immunity needs to be increased by vaccination. The age-specific seroprevalence rates are relatively similar among age groups with overlapping confidence intervals, therefore, the results would not have an influence on the vaccination strategy in terms of prioritizing age groups. Instead, the elderly are prioritized for vaccination on the basis of their higher risk of developing severe forms of COVID-19. However, significant differences in the seroprevalence among counties indicate that higher vaccination coverage will be needed in counties with lower seroprevalence rates, in order to prevent comparatively higher incidence in these counties in future epidemic waves.

The main advantage of the serological surveys is to assess the actual prevalence of the SARS-CoV-2 infection in the population and herd immunity, as well as to analyze the antibody level and duration of the immunity. However, some patients in the early acute phase of COVID-19 may be missed (serological "window" period). Molecular diagnostics (RT-PCR) in seronegative individuals allows additional prevalence estimates, including the acute infections without detectable antibodies [39]. In this study, data on the SARS-CoV-2 RT-PCR were not available, which should be pointed out and considered when interpreting the seroprevalence results, especially in seronegative individuals.

## 4. Materials and Methods

### 4.1. Study Population

**Figure 5:**
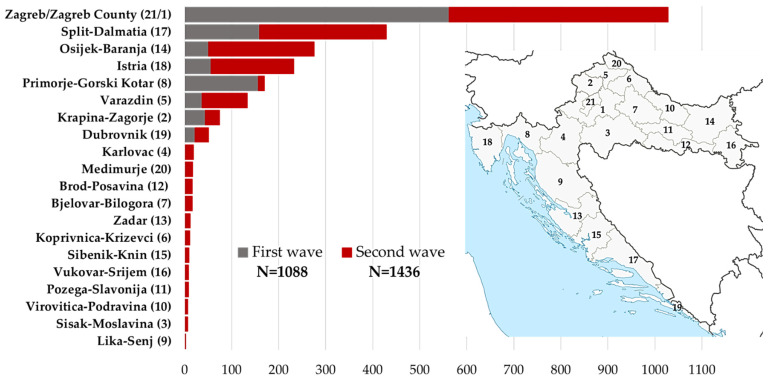
Geographic distribution of study participants.

A total of 2524 participants aged 9 months–83 years were included in the study. In the tested group, there were 993 (39.4%) males and 1531 (60.6%) females from all Croatian counties. Serum samples were collected at two intervals: I—1088 samples (at the end/after the first pandemic wave, May–July 2020); II—1436 samples (during/after the second pandemic wave, December 2020–February 2021). The distribution of samples according to counties is presented in Figure 5. 

### 4.2. Serology Testing

Initial serological screening (IgG antibodies) was performed using a commercial ELISA based on recombinant spike glycoprotein (S) and nucleocapsid protein (N) antigens of SARS-CoV-2 (ELISA COVID-19 IgG; Vircell Microbiologists, Granada, Spain). Results were expressed as antibody index; AI = (sample OD/cut off serum mean OD) × 10 and interpreted as follows: IgG < 4, negative, 4–6, borderline, >6, positive [40]. All reactive samples were further tested for confirmation using a virus neutralization test (VNT). The third passage of the SARS-CoV-2 strain, isolated in Vero E6 cells (ATCC CRL-1586) from a Croatian COVID-19 patient, was used as a stock virus. Virus titer (TCID_50_) was calculated using the Reed and Muench formula. An equal volume (25 μL) of serial two-fold dilutions of heat-inactivated serum samples (30 min/56 °C) and 100 TCID_50_ of SARS-CoV-2 were mixed and incubated at 37 °C with CO_2_ for one hour. Finally, 50 μL of 2 × 10^5^ Vero E6 cells/mL were added to each well. To ensure optimal testing results, the virus antigen used in each run was back-titrated, and a positive sample with a known titer as well as a negative control sample were included in each plate. The plates were incubated at 37 °C with CO_2_ and, starting from the third day, the plates were checked for the cytopathic effect. Titer was defined as the reciprocal of the highest serum dilution that showed at least 50% neutralization. NT antibody titer ≥ 8 was considered positive. VNT was performed in a biosafety level-3 facility [13].

### 4.3. Statistical Analysis

Seroprevalence rates by gender, age group and geographical region, and exact 95% confidence intervals (95% CI), were calculated using Stata 16. A Pearson’s chi-square test was used to compare the seroprevalence rates. Numerical variables are expressed as medians and interquartile ranges (IQR). A Kruskal–Wallis test was used to compare ordinal and continuous variables between multiple groups. The association between ELISA AI and VNT titer was assessed by Spearman’s rho rank-based correlation. The level of significance was set at α = 0.05.

## 5. Conclusions

The results of this first large seroprevalence study in the Croatian general population showed a significant increase in SARS-CoV-2 seroprevalence after the second pandemic wave. Although seropositive individuals were detected in all age groups, there was a significant difference in seropositivity among age groups and geographic regions. The age-associated prevalence was observed, with the lowest seroprevalence rates in the oldest and the youngest age groups compared to other groups. In addition, NT antibody titers seem to be age-related. In contrast to the seroprevalence, the highest NT activity was detected in the youngest participants and participants above 50 years. However, further studies are needed to confirm this observation.

## Figures and Tables

**Table 1:** Seroprevalence of SARS-CoV-2 in the Croatian population.

Demographic Characteristics	Tested N (%)	SARS-CoV-2 IgG ELISA ^a^		SARS-CoV-2 VNT ^b^	
		N (%)	95% CI	N (%)	95% CI
1st pandemic wave (N = 1088)					
Gender					
Male	371 (34.1)	12 (3.2)	1.7–5.6	1 (0.3)	0–1.5
Female	717 (65.9)	12 (1.7)	0.9–2.9	1 (0.1)	0–0.8
Age group					
<10 yrs	146 (13.4)	5 (3.4)	1.1–7.8	0 (0)	NA^c^
10–19 yrs	119 (10.9)	3 (2.5)	0.5–7.2	0 (0)	NA
20–29 yrs	117 (10.8)	0 (0)	NA	0 (0)	NA
30–39 yrs	241 (22.2)	3 (1.2)	0.3–3.6	1 (0.4)	0–2.3
40–49 yrs	130 (11.9)	5 (3.8)	1.3–8.8	1 (0.8)	0–4.2
50–59 yrs	114 (10.5)	2 (1.7)	0.2–6.2	0 (0)	NA
60–69 yrs	125 (11.5)	2 (1.6)	0.2–5.7	0 (0)	NA
70+ yrs	96 (8.8)	4 (4.2)	1.1–10.3	0 (0)	NA
Total	1088 (100)	24 (2.2)	1.4–3.3	2 (0.2)	0–0.7
2nd pandemic wave (N = 1436)					
Gender					
Male	622 (43.3)	156 (25.1)	21.7–28.7	123 (19.8)	16.7–23.1
Female	814 (56.7)	204 (25.1)	22.0–28.1	144 (17.7)	15.1–20.5
Age group					
<10 yrs	174 (12.1)	33 (19.0)	13.5–25.7	28 (16.1)	11.0–22.5
10–19 yrs	195 (13.6)	50 (25.8)	19.7–32.4	42 (21.5)	16.0–28.0
20–29 yrs	146 (10.2)	42 (28.9)	21.6–36.8	30 (20.5)	14.3–28.0
30–39 yrs	220 (15.3)	54 (24.6)	18.6–30.3	44 (20.0)	14.9–25.9
40–49 yrs	238 (16.6)	65 (27.4)	21.7–33.4	46 (19.3)	14.5–24.9
50–59 yrs	207 (14.4)	54 (26.2)	20.2–32.6	40 (19.3)	14.2–25.4
60–69 yrs	162 (11.3)	46 (28.6)	21.6–36.0	26 (16.0)	10.7–22.6
70+ yrs	94 (6.5)	16 (15.7)	10.0–26.2	12 (12.8)	6.8–21.2
Total	1436 (100)	360 (25.1)	22.8–27.4	268 (18.7)	16.7–20.8

^a^ ELISA = enzyme-linked immunosorbent assay; ^b^ VNT = virus neutralization test; ^c^ NA = not applicable.

**Table 2:** SARS-CoV-2 seroprevalence rates by county.

Region	N Tested	SARS-CoV-2 IgG ELISA		SARS-CoV-2 VNT	
		N Positive (%)	95%CI	N Positive (%)	95%CI
Zagreb + Zagreb County (21 + 1) *	460	94 (20.4)	16.8–24.4	70 (15.2)	12.2–18.8
Split-Dalmatia County (17) **	265	76 (28.7)	23.3–34.5	62 (23.4)	18.4–29.0
Osijek-Baranja County (14) *	225	72 (32.0)	25.9–38.5	51 (22.7)	17.4–28.7
Istria County (18) **	178	29 (16.3)	11.2–22.5	16 (9.0)	5.2–14.2
Varazdin County (5) *	97	35 (36.1)	26.6–46.5	24 (24.7)	16.5–34.5

* Continental; ** coastal.

## Data Availability

Not applicable.
